# Supplementary material for: Are carbon emissions trading and green financial instruments synergistic? -Comprehensive quantitative research based on content analysis
Source: PLoS One. 2024 Mar 7;19(3):e0298601. doi: 10.1371/journal.pone.0298601 (PMC10919601; doi:10.1371/journal.pone.0298601)
Supplement: S1 Data — (ZIP) [file pone.0298601.s001.zip › Data/地级市Z_F工作报告.zip!/╔╜╢1⁄2/╢1⁄2╙¬╩╨/└┤╫╘╣1⁄2╓┌║┼í╛┬φ┐╦╩2╛▌═°í┐_2015.docx]

各位代表：

现在，我代表市人民政府向大会报告政府工作，请予审议，并请市政协委员和其他列席会议的同志提出意见。

一、2014年工作回顾

过去的一年，在市委的正确领导下，全市认真贯彻落实中央和省委、省政府决策部署，以开展党的群众路线教育实践活动为主线，牢牢把握“高境界、高标准、高起点、大作为”的工作总要求和“转方式、调结构、扩总量、增实力、上水平”的工作大局，积极应对困难挑战，奋力推动科学跨越，经济社会发展取得新成绩。

一是稳增长，经济保持平稳较快发展。初步统计，2014年全市实现生产总值3430.5亿元，同比增长10%。加强大项目建设，全社会固定资产投资2708.2亿元，增长16.1%。积极扩大消费，社会消费品零售总额659.1亿元，增长12.6%，物价总水平保持稳定。全力支持实体经济发展，保障企业生产经营，规模以上工业增加值增长11.4%，主营业务收入达到1.3万亿元，过百亿企业达到27家。强化财政金融支撑，实现公共财政预算收入206.2亿元，增长12.2%；金融机构各项存款余额3300.1亿元、贷款余额2600亿元，分别比年初增加452.1亿元、438.7亿元。

二是求突破，牵动全局的大事成效显著。扎实开展“三大专项行动”。城市公共安全提升年行动，强化政府和部门监管责任，持续深入企业和基层明查暗访，督促整改安全隐患；实施五大安全工程95个项目，深化油区安全综合整治，开展“六打六治”打非治违，安全生产形势总体稳定。生态环境整治提升年行动，实施119项治理工程，取缔“土小”污染企业270家，清理整顿环保违规项目511个，淘汰黄标车老旧车2.5万辆，节能减排完成年度任务，环境质量得到改善。乡村文明建设提升年行动，集中开展村庄清脏治乱，实施硬化、亮化、绿化、美化工程，建立起县区统管、城乡一体的环卫运行机制，在全省城乡环卫一体化群众满意度调查中获得第一名。积极推进“十件大事”。东营港北防波堤和三个突堤加快建设，疏港铁路正式奠基，兴港公路通车。东营港城起步区建设快速推进。广利港疏港道路实现路基贯通。胜利机场调整优化了航线航班，国产大飞机试飞基地建设全面开工。德大铁路全线贯通，广寿铁路开通运营，黄大铁路、济东高速公路建设顺利推进。市一中、二中、中专学校迁建一期工程和市人民医院急诊急救中心暨内科病房楼完成主体。东营综合保税区申报、东营农高区创建国家级农高区取得重大进展。扎实开展全国文明城市创建，深入实施公共环境、公共秩序、社区管理、社会文化环境、公共设施建设等提升工程，成功创建为全国文明城市。

三是调结构，发展方式加快转变。加快发展现代农业，实施了124个重点项目，完成投资74.8亿元；粮棉夺得丰产，农业园区和龙头企业加快发展，优势产业不断壮大，农产品质量安全水平稳步提升；完成河道治理等一批水利工程。“渤海粮仓”科技示范工程扎实推进。加快推进新型工业化，坚持改造提升主导产业与培育壮大新兴产业“双轮驱动”，实施了341个重点项目，完成投资737.3亿元，淘汰一批落后产能，高新技术产业比重达到34.6%、提高1.1个百分点。出台加快服务业发展的政策措施，实施了30个重点项目，完成投资118亿元，加快企业剥离非主营业务，服务业增加值增长9.2%、比重提高1.1个百分点。坚持创新驱动发展，实施省级以上科技计划项目89项，新增科技型企业92家，大学科技园、可持续发展研究院载体功能不断增强。全国质量强市示范城市通过预验收，国家石油装备产品、轮胎及橡胶制品质量监督检验中心获批成立，知识产权示范城市考核列全国地级市第二名。

四是抓统筹，城乡区域一体化发展迈出新步伐。稳妥推进新型城镇化，城镇化率达到64%。加快中心城规划建设，严格规划审批执行，实施了47个城建项目、110项工程，生态新城等片区开发加快推进，科技馆等建成投用，11个片区改造稳步推进，东城商贸城综合整治基本完成，西城改造成效显著。加快城乡绿化美化，新增造林12.1万亩，圆满承办了省第五届城市园林绿化博览会。统筹推进城镇和农村社区建设，启动城市社区规范化建设，新建续建农村社区54个，黄河南展区建成143栋安置房。胜利油田坚持创新转型、从严管理，改革发展取得新成绩。市和油田坚持联席会议制度，合作建设特色产业园，共同推进城市和基础设施建设，统筹改善民生，油地共建开创新局面。中国石油大学胜利学院主校区正式启用并按新体制招生。军地团结共建、协同发展迈出新步伐。各县区、市属开发区经济实力明显增强。

五是促改革，发展动力活力进一步增强。重点改革实现突破。改革工商登记制度，新增私营企业6360家、增长91%，各类市场主体达到10.3万户。深化财税体制改革，开展专项资金清理，“营改增”试点稳步推进。加快金融改革创新，开业银行达到26家，休斯顿石油装备交易中心、华东石油交易中心开业运营；制定出台防范化解措施，维护良好的金融生态环境。加快农村产权制度改革，在全省率先完成农村土地承包经营权确权登记颁证工作，农村土地流转积极有序推进。加快未利用地开发利用改革试点。出台深化教育领域综合改革意见，稳步推进办学体制、考试招生等改革。继续深化医药卫生体制改革，第二批县级公立医院综合改革试点扎实推进。大力推进行政管理体制改革，精简市级行政审批事项119项，食品药品工商质监、卫生计生体制改革全面完成，自然保护区管理体制进一步理顺。进一步扩大对外开放，实现进出口132.6亿美元，其中出口60.9亿美元，分别增长4.1%和5.1%，高于全国、全省平均水平；实际利用外资2.2亿美元，增长13.8%；境外投资增长98.9%，对外工程营业额4.3亿美元。对口支援工作取得新成绩。

六是惠民生，群众得到更多福祉。新增城镇就业4.5万人，农村劳动力转移就业4.1万人，新增省级创业服务园区3个，胜利大学生创业园投入运行。城镇居民人均可支配收入36940元、增长8.7%，农民人均纯收入14456元、增长11.2%。完善社会保障体系，提高城乡居民医疗保险财政补助、基本养老保险、城乡低保、农村五保供养标准和企业退休人员待遇，建立城乡居民临时救助制度、大病保险制度和城镇职工长期医疗护理保险制度，保障水平居全省前列。建设各类保障性住房和棚户区改造安置房2.1万套。启动政府购买养老服务试点。民生实事完成年度计划。优先发展教育事业，小班化、集团化试点有序推进，东营区、河口区、垦利县通过义务教育发展基本均衡县区国家认定，东营职业学院通过国家骨干院校验收。健全公共卫生服务体系，人均基本公共卫生服务项目补助经费标准提高到35元，低生育水平保持稳定。深入实施文化惠民工程，成为全省首批公共文化服务标准化试点地区。全民健身公共服务体系进一步完善，竞技体育取得新成绩。强化食品药品监管，基本建立起覆盖城乡的四级安全监管网络。国防动员、民兵预备役建设、双拥共建和优抚安置工作不断加强。创新社会治理，改进信访工作，完善治安防控体系，社会保持和谐稳定。

七是转作风，政府自身建设不断加强。扎实开展党的群众路线教育实践活动，聚焦“四风”问题，大力正风肃纪，解决了一批群众反映强烈的突出问题,作风建设取得明显成效。严格落实中央八项规定精神，全面清理和处置违规公务用车、超标办公用房、“吃空饷”人员，大力压减“三公”经费支出，着力治理文山会海，政风和社会风气发生明显变化。自觉接受人大及其常委会法律监督、工作监督和政协民主监督，推行权力清单制度，建立政府法律顾问制度，强化行政监察和审计监督，依法行政水平明显提高。强化政府工作人员反腐倡廉教育，加强机关规范化建设，加大“庸懒散”治理力度，廉政勤政建设明显加强。

一年来成就的取得，是在中央、省委省政府和市委正确领导下，全市上下、油地军校、社会各界团结奋斗、携手共建的结果。在此，我代表市政府，向辛勤劳动的广大干部群众，向积极支持政府工作的人大代表、政协委员和社会各界人士，向所有关心支持东营发展的同志们、朋友们，表示崇高的敬意和衷心的感谢!

必须清醒地看到，我市发展中还存在不少矛盾和问题。受国内外经济大环境和油价大幅下滑影响，经济下行压力加大，部分企业生产经营困难，进出口受阻，财政收支矛盾突出，就业困难加剧；产业结构调整进展较慢，资源利用效率偏低，自主创新能力不强；部分农业基础设施老化，抵御自然灾害能力仍然较弱，农业综合效益不高；社会管理还存在薄弱环节，涉及群众切身利益的食品药品安全、生态环境、上学就医等方面仍有诸多不满意的地方，各种不稳定不安全因素仍然较多；部分政府工作人员法治意识比较薄弱，作风建设还需加强。我们一定高度重视人民群众的关切，直面自身工作中的问题，采取更有针对性的措施，认真加以解决。

二、2015年工作总体部署

我国经济发展已经进入新常态，我们必须深刻把握经济发展内涵、动力、格局的新变化，正确认识新常态，主动适应新常态，积极引领新常态。今年我们要深入学习贯彻党的十八大和十八届三中、四中全会和习近平总书记系列重要讲话精神，坚持稳中求进工作总基调，保持发展思路稳定性和工作连续性，以深入实施黄蓝国家战略为统领，以提高发展质量效益为中心，以全面深化改革为动力，着力加快结构调整，着力推进科技创新，着力改善生态环境，着力防范化解风险，着力推进依法行政，着力保障改善民生，完成“十二五”发展目标，规划“十三五”发展任务，努力实现经济持续健康发展和社会和谐稳定，以新的更大作为开创东营科学跨越新局面。

今年全市经济社会发展的预期目标是：生产总值增长9%左右，全社会固定资产投资增长15%左右，社会消费品零售总额增长12.5%左右，进出口总值、吸收外商直接投资保持适度增长，公共财政预算收入增长9.5%左右，城镇和农村居民人均可支配收入分别增长9.5%左右、10%左右，城镇登记失业率控制在3%以内，居民消费价格涨幅控制在3%左右，人口自然增长率控制在9.2‰以内，完成省政府下达的节能减排约束性目标。

实现上述目标，推动经济持续健康发展，必须增强消费、投资、出口均衡拉动作用。发挥消费的基础作用，努力增加城乡居民收入，加快发展现代流通方式，营造安全放心消费环境，积极扩大消费需求。发挥投资的关键作用，加快实施大项目带动战略，市级重点抓好92个重大项目建设，各县区、市属开发区分别抓一批重大项目，引导资金重点投向社会民生、农林水利、能源交通、生态环保、先进制造业、现代服务业等领域，提高投资质量。发挥出口的支撑作用，提升产品国际市场竞争力，培育新的出口增长点。

必须保障企业健康运行。企业是经济之源、发展之本。面对复杂严峻的经济形势，我们要把保障企业健康运行作为今年经济工作的重要任务，继续实施市级领导联系重点企业和驻企联络员制度，着力在企业融资、市场开拓、技术改造、减轻负担方面做好工作、搞好服务，引导企业增强应对困难和挑战的坚定信心，奋力渡过难关。鼓励引导银行、保险、证券等金融机构加快产品和服务创新，服务实体经济发展。加快组建由政府出资控股参股的大型融资担保公司，完善银担合作机制，进一步撬动金融机构的信贷投放。发挥应急转贷资金服务功能，保障企业资金链安全。深入实施“137”计划，培育一批支撑力和带动力强的“台柱子”企业。大力发展民营经济，加大中小微企业扶持力度，培育新的经济增长点。重视企业家队伍建设，培养一批引领产业发展、守法诚信经营、履行社会责任的优秀企业家。

必须持续深化“三大专项行动”。一是城市公共安全专项行动。全面推行安全生产网格化管理，层层落实各方责任，强化网格内单位管控，真正形成“全覆盖、无缝隙”的责任体系。继续抓好油气管道占压、道路交通、建筑施工、消防、校车等行业领域隐患整治，实施一批公共安全保障项目，加大明查暗访力度，保持打非治违高压态势，预防和减少各类生产安全事故。二是水气污染整治专项行动。实施中心城主要河流污染综合治理工程，全面整治老广蒲沟、新广蒲河、五干排、六干排、五六干合排、广利河、东营河等市控以上重点河流，完善雨污管网和截污管网，彻底取缔污水直排口，省控重点河流基本消除劣五类水体。加快推进燃煤电厂脱硝设施建设，抓好石化企业挥发性有机物、城市扬尘、黄标车淘汰等专项治理，进一步提升空气质量。严格落实环保属地管理责任，加强环保执法队伍和监测能力建设，坚决取缔“土小”污染企业，全面实施行政执法与刑事司法联动，重拳打击违法排污行为。利用三年左右时间，使东营的水气质量进入全省第一方阵。三是乡村文明建设专项行动。继续抓好农村硬化、绿化、亮化、美化，扩大环卫一体化覆盖面，搞好秸秆气化、农村社区污水处理、改厕试点。加强农村社区服务中心建设，围绕群众生产生活需求，完善服务功能，统一规范标准，真正发挥服务群众作用。开展美丽乡村连片创建示范行动，提升农村环境面貌和群众生活品质。

必须推进区域协调发展。加强油地军校协同共建，突出项目引领，加快合作事项推进，围绕土地利用、矿产开发、城市建设、安全环保、民生改善等领域，共同实施一批新的合作项目；完善共建机制，搭建决策层对话磋商、管理层联合办事、执行层联手落实的平台，提高各层面的执行效率，合力推进工作，共建美好家园。支持胜利油田积极应对低油价，转型发展创效益。大力培育各具特色的经济板块，鼓励各县区、开发区加快产业转型升级、深化改革开放、加强科技创新，支持利津县加快发展、跨越赶超。

三、加快产业转型升级

推动产业转型升级是决定东营前途命运的战略任务，必须以壮士断腕的决心和勇气，坚决打好这场硬仗。

加快推进新型工业化。重在提升产业层次，以增量优化促转调，提高质量效益。主导产业抓升级，以产品高端化、装备高端化、市场高端化为重点，着力打好“高端提升”攻坚战，提高产业附加值和产品市场竞争力。石油化工产业，坚持园区化集中布局，通过引进高新技术装备衍生一批项目，推动产品质量升级，努力向高端精细化工领域延伸。橡胶轮胎产业，按照国际先进的产业标准和工艺，提升装备水平，调整产品结构，加快向中高端市场迈进。石油装备产业，加强科技创新和品牌建设，加快开发核心技术和高附加值产品，逐步实现由卖产品向卖技术、卖服务转变。新兴产业抓壮大，围绕汽车及零部件、新能源、新材料、新医药等重点领域，加快推进在建项目建设，围绕产业链引进实施一批新项目，尽快形成规模优势。推动工业化和信息化深度融合，促进设计研发信息化、生产过程智能化、生产装备数字化、经营管理网络化。深入实施质量强市战略，发挥国家石油装备产品、轮胎及橡胶制品、石油炼制产品质量监督检验中心作用，加强质量、标准和品牌建设。

提升服务业规模水平。重在扩大总量，优化结构。全面落实促进服务业发展的政策措施，实施一批重点项目，比重提高1个百分点。大力发展生产性服务业。继续做好工业企业非主营业务剥离，支持现代物流、研发设计、检验检测、云计算、服务外包、会展等重点领域发展，促进服务业和制造业融合协调发展。做大做强金融业，加快金融集聚区建设，引进各类金融机构，完善金融服务体系；推进大宗商品交易场所加快发展；大力发展多层次资本市场，支持企业上市、发行公司债券、在银行间市场筹集资金；支持金融机构参与信贷资产证券化试点；规范发展民间金融组织，强化地方金融监管，打击恶意、违法追讨债务行为；建章立制，多措并举，防范化解金融风险。加快发展生活性服务业。培育电子商务、文化创意、动漫游戏等新兴业态，壮大文化旅游、健康养老等产业。提升重点旅游景区服务接待能力，积极发展乡村旅游，推进旅游业转型升级。统筹规划养老服务设施，支持社会力量举办养老机构，提高养老服务业发展水平。促进房地产业健康发展。

深入实施创新驱动发展战略。着力培育创新型企业。实施创新型企业培育计划，引导大企业加强自主创新能力建设，支持中小企业加强产学研结合，争创软件火炬特色产业基地，高新技术产业产值达到5000亿元。着力发展科技园区。加快推进省级服务业综合改革试点，提升大学科技园建设水平；以胜利科技创新园区为载体，打造高端石油装备创新创业基地；加强县域科技园区建设，打造创新创业聚集区。着力增强科技研发能力。加强可持续发展研究院创新能力建设，抓好国家采油装备工程技术研究中心建设，加快推进国家电子陶瓷材料工程中心和有色金属、绿色化工产业技术研究院等平台建设。着力改革科技体制机制。深化科技金融结合，积极开展科技创投工作；加强与高校院所合作，推进协同创新。人才是创新驱动的核心要素,深入实施“黄河三角洲学者”、“金蓝领”培训等重点人才工程，完善人才引进、培养、使用和激励机制，吸引高端人才聚集。加强知识产权运用和保护，积极推进国家知识产权示范城市建设。

强化节能环保硬约束。加强源头控制，严把新上项目能评、环评关口，全面提高准入门槛。下决心淘汰落后产能，该关停的坚决关停，该淘汰的坚决淘汰。实行能源消费强度和总量双控制，强化重点领域、重点行业、重点企业节能降耗。搞好餐厨等废弃资源综合利用。做好中美低碳生态城市试点示范工作。加强湿地生态保护。加强造林绿化，实施重点干线公路、高标准农田林网、村庄绿化等工程，启动中心城城北防护林工程，抓好生态林场建设，有序实施城市道路及裸露地面绿化，积极创建绿化示范村镇、绿化模范县、森林城市。

四、全面加强“三农”工作

“三农”问题始终是事关全局的重大问题,也是推进科学跨越面临的重点难点问题。要以建设新农村为统揽，以农民增收为核心,以产业发展为基础，加快农业现代化，推动农业转型发展。

提升现代农业发展水平。围绕畜牧、渔业、食用菌等优势产业，推广应用农业新技术、新品种、新工艺，大力发展设施农业、工厂化农业，加快产业转型升级。规划建设一批集中连片的农业综合园区，制定标准规范，创新种养模式、管理模式，提升园区质量效益。突出抓好东营农高区建设，聚集各种资源要素，实施好研发总部基地等平台和产业项目，争取获批国家级农业高新区。高度重视粮食生产，实施好粮食高产创建、“渤海粮仓”科技示范工程和耕地质量提升计划。支持现代畜牧业示范区、现代渔业示范区加快发展，推进“海上粮仓”建设。

培育农业新型经营主体。重点扶持规模大、管理规范、带动能力强的20家龙头企业、50家农民合作社、50个家庭农场加快发展，促进一二三产融合，示范引领农业产业化、规模化水平提升。支持骨干龙头企业发展农产品精深加工和生态循环农业，打造全产业链条。推动农业龙头企业集群发展，密切与农户、农民合作社的利益联结关系。加强宣传推介、品牌培育、市场建设，着力提高黄河口名优农产品市场占有率。

加强农业农村基础设施建设。深入实施农田水利基本建设五年规划，重点实施36个项目，改善扩大灌溉面积70万亩、除涝面积93万亩，发展节水灌溉面积10.5万亩。继续抓好农村饮水安全，规范工程管理，提高水质合格率。新建改造农村公路500公里，改造危桥10座。启用国家中心渔港，抓好南水北调配套工程建设和农村电网续建配套。

深化农村综合改革。有序开展集体土地、荒地、滩涂、养殖水域、林地、草场等使用权确权颁证，赋予农民更多财产性权利。建立农村产权交易市场，促进农村生产要素优化配置。扎实开展新型农村合作金融试点工作，规范发展农民专业合作社信用互助业务，探索农村承包土地经营权和农民住房财产权等各种农村产权的流转、抵押、担保办法，设立“三农”融资性担保公司，改善新型农业经营主体融资环境。推进供销社综合改革。

五、积极推进新型城镇化

把新型城镇化作为转方式调结构的重要引擎，坚持以人为本、产城融合、注重质量，统筹推进城乡建设，促进一体化发展。

提升中心城规划建设管理水平。按照黄河三角洲中心城市的定位，突出大空间、大绿地、大水面、大湿地的特色，加快建设秀美宜居的现代化城市。高标准编制各类规划，防止盲目铺摊子，提高城市建设集约化水平。加强规划管理，严格规划审批执行，坚决防止随意改变容积率等行为，维护规划的科学性、严肃性和权威性。西城重点实施旧片区开发改造，东城重点完善功能、提升形象，在加快推进生态新城、金湖银河片区、森林公园等续建工程的同时，围绕市政设施、城市道路、地下管网、污染治理、园林绿化等启动实施一批新建项目。加强园博园管理提升，打造城市特色景区。巩固创建全国文明城市成果，理顺中心城管理体制，提高管理成效。深入推进智慧城市建设，重点抓好市政设施智能化建设，实现数字化城市管理全覆盖。积极探索市政管养市场化运作模式，提高市政设施管养水平。

统筹推进县城、小城镇和社区发展。提升县城建设水平，增强县域中心城市支撑作用。依托省级示范镇、国家级改革试点镇和国家重点镇，加快小城镇建设。扎实推进城市社区规范化建设，提高网格化、信息化管理水平。加快新型农村社区建设，稳妥实施城中村、城边村、空心村搬迁改造和乡镇驻地村整体改造，完善基础设施和公共服务设施。争取国家资金支持，稳妥推进黄河南展区新型农村社区建设。加快推进城镇一体化管理，提高管理水平。

加强重大基础设施建设。东营港完成北防波堤和一港池航道防护工程，建成启用三突堤6个码头。加快推进港城及疏港铁路建设。广利港疏港道路、海红路改造工程建成通车，展开码头和物流园区吹填建设。加快建设国产大飞机试飞基地，启动跑道延长和飞行区配套升级改造工程，适时增开航线航班。东营火车南站竣工投用，推动德大铁路通车运营，加快济东高速公路建设，积极推进黄大铁路、淄东铁路建设改造。加快推进大唐东营电厂建设。

六、全面深化改革开放

推动东营科学跨越，活力之源在于改革开放。我们必须坚定不移推进改革开放，以改革增活力，以开放强动力。

深化重要领域和关键环节改革。（1）完善现代市场体系。继续深化工商登记制度改革，进一步放宽市场主体准入条件。完善主要由市场决定价格的机制，推进公用公益领域价格改革，放开竞争性环节价格，深化水、电、气等资源价格改革。探索建立城乡统一的建设用地市场，综合运用未利用地开发试点、增减挂钩等政策，增强用地保障服务能力。（2）深化财税体制改革。加快预算编制管理改革，全面推行预决算公开。推进财政支出管理改革创新，减少和取消财政对产业、企业的直接补助，积极发展各类政府引导基金、创业投资基金，带动先进制造业和新兴服务业发展;推进PPP模式应用和政府购买服务等方式，拓宽城市公共设施融资渠道，鼓励社会资本参与污水垃圾处理等设施建设经营;盘活财政存量资金，提高使用效益;树立过紧日子思想，从严控制一般性支出。完善政府债务管理机制，切实防范和化解债务风险。（3）深化行政体制改革。坚持简政放权、放管结合，继续取消和下放一批市级行政审批事项，提高行政审批服务效能，加大事中事后监管力度。加快建立“四清单一平台” 政府权力运行体系。扎实推进政府机构改革，优化政府机构设置和职能配置。深化事业单位改革，基本完成承担行政职能的事业单位改革任务。按期完成公车改革。

推进更高水平的对外开放。坚持以开放的眼光、开放的胸襟、开放的谋略谋划发展，全面推进对外开放。支持企业拓展境外营销网络，主动应对国际贸易摩擦，确保出口稳定增长。加快外贸出口基地建设，加大商标国际注册力度，培育国际知名品牌，扩大自有品牌产品出口。积极支持企业扩大进口。加强海关综合监管区和电子口岸建设，全面推广关检合作，建立“三个一”通关模式。推行主题招商，引导外资参与产业转型升级。推进东营港码头对外开放，抓好东营综合保税区、中美清洁能源合作产业园、中韩产业园建设。扩大对外开放领域，积极推进服务业、社会事业、重大基础设施、农业等领域开放。全面推进对外交流合作，加强对自由贸易区政策的研究，引导企业积极参与“一带一路”和东亚海洋合作平台建设，加快“走出去”步伐。加强与京津冀、长江经济带交流合作，实现资源共享、优势互补、互利共赢。

七、持续保障改善民生

善政之要，惟在养民。我们一定要优先保障民生投入，优先安排民生项目，优先解决民生问题，让发展成果更多更公平惠及全市人民。

努力扩大就业。重点抓好高校毕业生就业和化解过剩产能中出现的下岗再就业工作，统筹抓好农村转移劳动力、城镇困难人员、退役军人就业。新开发公益性岗位1000个以上，新建就业见习基地20处，开展职业技能培训4万人次。发挥小额担保贷款促进就业创业的作用，激励高校毕业生和广大青年、妇女创业。探索建立调查失业率制度。组织实施农民工“三项行动计划”，建设中心城区农民工综合服务中心，落实农民工工资支付“一书两金一卡”制度，依法及时打击欠薪行为，保障农民工合法权益。

完善社会保障体系。城乡居民医疗保险个人缴费标准统一调整为每人每年200元，建立按病种分值结算为主的付费机制。实施机关事业单位养老保险制度改革，完善被征地农民养老保险政策，推行基本殡葬免费服务。建立社会救助联动平台，实行社会救助“一门受理、协同办理”。实施残疾人“整体赶平均、共同奔小康”行动。建设市救灾物资储备管理中心。

坚持教育优先发展。深化教育领域综合改革，完善教育经费保障机制和教育人事管理制度，改革创新办学体制、资源配置方式、人才培养模式，稳步实施考试招生和评价制度改革。扎实推进第二轮学前教育三年行动计划，加快市一中、二中、中专学校迁建一期工程和城区学校、幼儿园建设，推动广饶县、利津县通过义务教育发展基本均衡县区国家认定。全面实施素质教育，深化基础教育课程改革，促进小班化教育内涵发展和学校特色发展。积极发展民办教育。加强教师队伍建设。深入推进油地校合作共建中国石油大学胜利学院，巩固和扩大东营职业学院国家骨干院校建设成果。

保障人民群众健康。以公立医院改革为重点，继续深化医药卫生体制改革。加快市人民医院急诊急救中心暨内科病房楼建设。深入推进医疗、医保、医药三医联动，统筹推进相关领域改革。实施基层医疗卫生服务能力提升工程，推进乡村卫生服务一体化，积极推行家庭医生式服务，稳定优化乡村医生队伍。加快推进公共卫生服务体系建设，实施国家基本服务项目，提高人均经费标准，推动公共卫生服务均等化。加强疾控机构规范化建设，建成投用市疾控中心检验综合楼，提升重大疾病防控水平。深入开展爱国卫生运动。以稳定低生育水平为中心，强化基层基础，促进人口长期均衡发展。

推动文化发展繁荣。围绕实现“中国梦”开展社会主义核心价值观宣传教育，加强“四德工程”建设，提高全社会文明程度。实施文化惠民工程，创建成为省级公共文化服务体系示范区，争创国家级示范区。积极开展省级公共文化服务标准化试点，配套完善文化服务设施，加强文艺人才队伍建设。加强网络监管，倡导健康文明网络文化。广泛开展全民健身运动，办好黄河口国际马拉松赛。

加强食品药品安全监管。健全食品药品监管网络体系，加快市食品药品检验检测中心建设，建好县、乡、村（社区）三级执法监管队伍，整合检验检测资源，持续开展专项整治。加强农产品质量安全监管，建立主要农产品标准体系,逐步建立农产品质量安全可追溯制度，坚决杜绝不合格农产品进入市场，确保人民群众“舌尖上的安全”。

创新社会治理。加强法治宣传教育，开展多形式法治创建活动，增强全民法治观念，夯实法治社会基础。引导社会组织健康发展，鼓励和支持社会组织参与社会治理和公共服务。完善法律援助制度，健全司法救助体系，推动公共法律服务向基层延伸。加强人民调解和社区矫正规范化建设，完善对流动人口、特殊人群的服务管理。加强和改进信访工作，健全矛盾纠纷排查化解机制，及时就地解决群众合理诉求。深入推进平安东营建设，完善社会治安综合防控体系，加强油区安全综合整治，依法严密防范和惩治违法犯罪活动，严厉打击欺行霸市、强揽工程等不法行为，确保社会秩序持续稳定。

办好民生实事：（1）提高城乡居民社会保障水平，居民基本医疗保险财政补助标准提高到每人每年400元，提高居民养老保险缴费补贴，城市低保标准每人每月提高30元，农村低保标准每人每年提高360元，农村五保供养标准每人每年提高500元。（2）建设保障性住房和棚户区改造安置房7551套。（3）为城乡困难老年人购买养老服务。（4）完善中心城智能交通及安全设施。（5）提升校园及校车安全监管水平。（6）更新环保型公交车120辆,改造候车亭120个。（7）深化新型农民学校工作，实施农民工职业技能提升行动。（8）提高食品安全抽检频率。（9）建设网上远程医疗服务系统。（10）推行公共自行车系统试点。对民生实事，我们要严格落实责任制，强化督查调度，加快推进实施，确保按期完成。

八、以改革创新精神推进政府自身建设

面对艰巨繁重的改革发展任务,必须加快转变政府职能，建设职能科学、权责法定、执法严明、公开公正、廉洁高效、守法诚信的法治政府和服务型政府。

深入推进依法行政。认真贯彻依法治国基本方略，全面推进法治东营建设。坚持依法科学民主决策，把公众参与、专家论证、风险评估、合法性审查、集体讨论决定确定为重大行政决策法定程序。严格执行重大决策终身责任追究制度及责任倒查机制。坚持执法为民，严格规范公正文明执法。各级政府及工作人员要带头遵守宪法和法律，提高法治意识和依法行政能力。全面推进决策公开、执行公开、管理公开、服务公开、结果公开，让人民监督权力，让权力在阳光下运行。坚决执行人大及其常委会决议决定，自觉接受法律和工作监督，支持政协参政议政，主动接受政协民主监督，广泛听取民主党派、工商联、无党派人士和人民团体意见，更加重视接受群众监督和舆论监督。加强审计、监察专门监督和政府层级监督，做到行政权力运行到哪里，行政监督就跟进到哪里。

持续加强作风建设。巩固群众路线教育实践活动成果，深入落实中央八项规定、省委实施意见和市委实施办法，驰而不息地整治“四风”,实现作风建设制度化、常态化。牢记群众观点，践行群众路线，领导干部带头深入基层调查研究,倾听群众呼声,帮助解决问题。大力弘扬求真务实优良传统，精简会议文件，改进文风会风,坚决不搞形象工程、政绩工程。加强政府绩效管理,深化“庸懒散”专项治理。倡树正气正劲，激励干事创业，凝聚起推动科学跨越的强大正能量。发扬“钉钉子”精神, 以踏石留印、抓铁有痕的劲头抓好工作落实。全体政府工作人员要践行“三严三实”要求，牢记使命责任，勇于担当作为，为东营发展拼搏奋进，为百姓福祉甘于奉献。

坚持从严治政。认真贯彻落实中央和省、市委从严治党的部署要求，切实把从严治党要求落实到从严治政中，做到真管真严、敢管敢严、长管长严。加强制度建设，健全从严治政的制度体系，扎紧制度的笼子，坚持用制度管权管事管人。严明党纪政纪，违纪必究，失责必问，做到纪律面前人人平等，遵守纪律没有特权，执行纪律没有例外。严格落实党风廉政建设责任制，严格遵守廉洁从政各项规定，坚决查处违纪违法案件，以零容忍态度惩治腐败。严格执行厉行节约反对浪费条例，大幅压减行政成本，把节约的资金用到为民办实事好事上，让政府过紧日子、人民过好日子。

各位代表！新常态需要新作为，大干劲才能大发展。让我们紧密团结在以习近平同志为总书记的党中央周围，在省委、省政府和市委的坚强领导下，凝聚起全市人民的智慧力量，脚踏实地，苦干实干，改革创新，奋发有为，为推动东营科学发展新跨越而努力奋斗！
